# Multimorbidity in Diabetic Patients Admitted to a Tertiary Care Center: A Descriptive Cross-sectional Study

**DOI:** 10.31729/jnma.7922

**Published:** 2023-01-31

**Authors:** Tunam Khadka, Ganesh Kumar Giri, Deependra Mandal, Ashmita Shrestha, Alok Dhungel, Abhinav Vaidya

**Affiliations:** 1 Kathmandu Medical College and Teaching Hospital, Sinamangal, Kathmandu, Nepal; 2 Manmohan Memorial Medical College and Teaching Hospital, Swoyambhu, Kathmandu, Nepal; 3 Department of Medicine, Kathmandu Medical College and Teaching Hospital, Sinamangal, Kathmandu, Nepal; 4 Department of Community Medicine, Kathmandu Medical College and Teaching Hospital, Sinamangal, Kathmandu, Nepal

**Keywords:** *co-morbidity*, *diabetes mellitus*, *multimorbidity*, *osteoarthritis*

## Abstract

**Introduction:**

Multimorbidity is defined as the co-occurrence of two or more chronic conditions in the same individual. Type 2 Diabetes Mellitus rarely occurs without coexisting diseases. With an increasing elder population and longevity, elder adults have a higher prevalence of chronic morbidity, thus increasing the chances of experiencing more than one non-communicable chronic condition, where the impact of multimorbidity is greater than the cumulative effect of the single condition. The study aimed to find out the prevalence of multimorbidity in diabetic patients admitted to a tertiary care centre.

**Methods:**

A descriptive cross-sectional study was conducted utilising hospital records of patients with type 2 diabetes mellitus admitted to the Department of Medicine from 1 April 2021 to 1 April 2022. Ethical clearance was obtained from the Institutional Review Committee of the same institute (Reference number: 12082022/07). The diagnosed cases of type 2 diabetic patients aged more than 18 years and confirmed with serum glucose levels were included in the study. Convenience sampling was used. Point estimate and 95% Confidence Interval were calculated.

**Results:**

Out of the 107 diabetic patients, multimorbidity was present in 75 patients (70.10%) (61.4278.77, 95% Confidence Interval).

**Conclusions:**

The prevalence of multimorbidity is higher than the similar studies done in similar settings.

## INTRODUCTION

Multimorbidity is defined as the co-occurrence of two or more chronic conditions in the same individual^1^ and has been estimated to affect up to 95% of the primary care population aged 65 years and older.^2-3^

In Southeast Asia, 90 million people have diabetes and one person dies every five seconds due to its complications in the world. The common chronic disease in multimorbidity patients is stroke, cancer, ischemic heart disease, and chronic obstructive pulmonary disease (COPD).^4-5^ The census of Nepal shows an increasing trend of the geriatric population i.e. 1.5 million (6.5%) in 2001 which increased to 2.5 million (8.1%) in 2011.^6^ With the increase in longevity, the likelihood of experiencing more non-communicable chronic conditions also increases.^7-9^ The impact of multimorbidity is greater than the cumulative effect of the single condition. Type 2 Diabetes Mellitus (T2DM) rarely occurs without coexisting diseases.

This study aimed to find out the prevalence of multimorbidity among diabetic patients admitted to the medicine department of a tertiary care centre with T2DM.

## METHODS

A descriptive cross-sectional study was conducted in the Department of Medicine of Kathmandu Medical College and Teaching Hospital among patients diagnosed with T2DM and admitted from 1 April 2021 to 1 April 2022. Ethical clearance was obtained from the Institutional review committee of the same institute (Reference number: 12082022/07), and the data was collected from the medical record section. The diagnosed cases of type 2 diabetic patients aged more than 18 years and confirmed with serum glucose levels either of; fasting more than 126 mg/dl or postprandial more than 200 mg/dl or HbA1c more than 6.5% were included in the study. Patients with ages below 18 years, other than Type 2 diabetes, incomplete medical records and patients of other departments were excluded. A specially designed form was used to fill in the data. Minimization of bias was done as far as possible but still, biases like repetition and missing information may be there.

Diagnosed cases of Chronic Obstructive Pulmonary Disease (COPD) as documented using a bronchodilator, Domiciliary oxygen were included whereas for Cardiovascular Diseases (CVD) having HTN or using hypertensive medication and documented history of Heart attack, angina was included. Patients under maintenance haemodialysis (MHD) or diagnosed Chronic Kidney Disease (CKD) and diagnosed osteoarthritis (OA) along with self-reported chronic joint pain were included. The sample size was calculated using the formula:


$$\begin{array}{ll} \text{n} & ={\text{Z}}^{2}\times \frac{\text{p}\times \text{q}}{{\text{e}}^{\text{2}}}\\ & =1.96^{2}\times \frac{0.50\times 0.50}{0.10^{2}}\\ & =97 \end{array}$$

Where,

n = minimum required sample size
Z = 1.96 at 95% Confidence Interval (CI)
p = prevalence taken as 50% for maximum sample size calculation
q = 1-p
e = margin of error, 10%

The minimum required sample size was 97. However, the final sample size taken was 107.

Data were entered in a Microsoft Excel Version 2016 and analysed with IBM SPSS Statistics 20.0. Point estimate and 95% CI were calculated.

## RESULTS

Out of the 107 diabetes patients admitted in between the study period, multimorbidity was present in 75 (70.09%) (61.41-78.77, 95% CI) with a mean age of 66.71±13.02 years. Among them, males 34 (45.33%) and females were 41 (54.66%). Among the multimorbidity cases, DM with other 2 comorbidity 30 (40%) was the highest and lowest being DM with other four comorbidities one (1.33%). The patient with CVD was 68 (90.66%) which is the highest, followed by OA 32 (42.66%) then COPD 30 (40.00%) and the patient with CKD was found to be 18 (24.00%) being the least prevalent (Table 1).

**Table 1:** Multimorbidity and Comorbid condition (n = 75).

Morbidity	n (%)	Female n (%)	Male n (%)
Multimorbidity	75 (70.09)	41 (54.66)	34 (45.33)
No multimorbidity	32 (29.91)	8 (25)	24 (75)
Comorbidities			
DM with 1 comorbidity	24 (32)	13 (54.16)	11 (45.84)
DM with 2 comorbidities	30 (40)	20 (66.66)	10 (33.33)
DM with 3 comorbidities	20 (26.66)	8 (40)	12 (60)
DM with 4 comorbidities	1 (1.33)	-	1 (100)

The age distribution of the multimorbidity was more towards the older age group 60-80 years age group 47 (62.66%) followed by age 40-60 years 18 (24.00%) with least being age 19-40 years 4 (5.33%). Among them, males were 34 (45.33%) and females 41 (54.66%) showing female predominance. Eleven (14.66%) of them consumed alcohol and 20 (26.66%) of them were smokers. The mean hospital stay was found to be 6.31±4.97 days with the minimum hospital being one day and the maximum is 31 days (Table 2).

**Table 2:** Age and sex distribution (n= 75).

Age	^*^MM n (%)	^†^NO MM n (%)
20-40	4 (5.33)	9 (28.12)
41-60	18 (24)	21 (65.62)
61-80	47 (62.66)	2 (6.25)
81-100	6 (8.00)	-
Total	75 (100)	32 (100)
Sex		
Male	34 (45.33)	24 (75.00)
Female	41 (54.66)	8 (25.00)

* MM: Multimorbidity

† No MM: Non-Multimorbidity

## DISCUSSION

Our study demonstrated the high burden of non communicable disease (NCD) multimorbidity among type 2 diabetic people. The prevalence of multimorbidity among type 2 diabetic people in our study was 70.10%. A similar study done in the USA showed similar multimorbidity (74.0%),^n^ among diabetic patients. However, a similar study among the general public through random sampling was quite low. The finding is much higher than the rate reported in South Asian countries, India and Pakistan (9.4%)^12^ and Bangladesh (8.4%). The prevalence of chronic conditions tends to differ across different countries owing to differences in dietary practices, lifestyle, alcohol consumption, and tobacco use. Hence it is not uncommon for multimorbidity to vary across countries.

Our study also showed female preponderance similar to other studies.^13^ Ageing leads to a decrease in physiologic function giving rise to multiple comorbidities.^14^ This may be due to age being a non-modifiable risk factor for non-communicable diseases.

Smoking and alcohol consumption was less than in other similar studies.^15^ Many higher studies show that alcohol and smoking are major causes and risk factors of Non-communicable multimorbidity conditions.^16-17^ Patients are often advised to cease smoking and alcohol to prevent the comorbidities using it as a risk factor tool for NCD. However, in our study, there was low smoking and alcohol consumption in contrast to a high multimorbidity prevalence. Such findings might be due to the cessation of smoking and alcohol by patients after diagnosis of at least one NCD.

The most common comorbidities were CVD 68 (90.7%), followed by OA 32 (42.66%) then COPD 30 (40.00%), and CKD 18 (24.00%) being the least. The occurrence of CVD as the most common comorbidity may be due to diabetes and hypertension, a component of CVD; sharing the common modifiable risk factor. Such findings bring a challenge in providing appropriate care to patients due to the lack of guidelines for multimorbidity. Facing challenges in terms of sampling in multimorbidity also plays a role in the lack of evidence-based medication for such patients.^19^

The limitation of our study is that it cannot be generalised, as it is done at the tertiary level with a low sample size. A further higher study needs to be done for data on the population. The mentioned record might also be biased due to the mishandling of records.

## CONCLUSIONS

The prevalence of multimorbidity is higher than in similar studies done in similar settings. In the Nepalese context, patients with multimorbidity rely on specialist services at secondary or tertiary hospitals as the primary healthcare system does not have the capacity to adequately assess and manage non-communicable chronic conditions, including multimorbidity. There is a need for a multisectoral integrated primary care approach. Further study is required with a larger sample size and probability sampling method for better scientific findings. It will help concerned authorities to provide guidelines for medical practitioners in the decision-making of these patients.
